# Production of *Beauveria bassiana* Fungal Spores on Rice to Control the Coffee Berry Borer, *Hypothenemus hampei*, in Colombia

**DOI:** 10.1673/031.008.4101

**Published:** 2008-03-21

**Authors:** Francisco J Posada-Flórez

**Affiliations:** Centro Nacional de Investigaciones de Café (Cenicafé). A. A. 2427 Manizales. Caldas, Colombia

**Keywords:** spore harvesting, germination, pathogenicity, broca

## Abstract

Two isolates of fungal entomopathogen *Beauveria bassiana* (Balsamo) Vuillemin (Hypocreales: Clavicipitaceae) were grown on cooked rice using diphasic liquid-solid fermentation in plastic bags to produce and harvest spore powder. The cultures were dried and significant differences were found for isolates and time of harvest. The spores were harvested manually and mechanically and after the cultures were dried for nine days, when moisture content was near 10%. After harvesting, spores were submitted to quality control to assess concentration, germination, purity, moisture content, particle size and pathogenicity to the coffee berry borer, *Hypothenemus hampei* (Ferrari) (Coleoptera: Curculionidae). Spore productivity on cooked rice was less than 1×10^10^ spores/g using both manually and mechanically harvesting methodologies. Germination at 24 hours was over 75% and pathogenicity against *H. hampei* was over 92.5%. This methodology is suitable for laboratory and field studies, but not for industrial production when a high concentration of spores are required for formulation and field applications.

## Resumen

Dos aislamientos del hongo entomopatógeno *Beauveria bassiana* (Balsamo) Vuillemin (Hypocreales: Clavicipitaceae) se cultivaron en arroz cocido usando el método de fermentación difásico liquide — sólide en boisas plásticas para producir y cosechar las esporas. Los cultives se secaron y se encontraron diferencias significativas para los aislamientos y el tiempo de cosecha. Las esporas se cosecharon manual y mecánicamente después de secar los cultives por nueve dias, cuando el contenido de humedad estuvo cerca del 10%. Después de la cosecha de las esporas, éstas se sometieron a control de calidad donde se evaluó concentración, germinatión, pureza, contenido de humedad, tamaño de particula y patogenicidad sobre la broca del café, *Hypothenemus hampei* (Ferrari) (Coleoptera: Curculionidae). La producción de esporas en cultives de arroz cocido presentó valores menores de 1×1010 esporas/g para ambas metodologías de cosecha. La germinación presentó valores superiores a 75% a las 24 horas y la patogenicidad estuvo sobre 92.5%. Esta metodología de producción del hongo *B. bassiana* es adecuada para estudios de laboratorio y campo, pero no para la producción industrial donde se requiere alta concentración de esporas para la formulación y la aplicación.

## Introduction

The coffee berry borer, *Hypothenemus hampei* (Ferrari) (Coleoptera: Curculionidae) poses a serious threat to coffee production throughout the world due to its destruction of the coffee seed. Among the various biocontrol methods used to control this insect, the fungal entomopathogen *Beauvena bassiana* (Balsamo) Vuillemin (Hypocreales: Clavicipitaceae) is highly promising. The fungus has been shown to cause high mortality in various countries where the coffee berry borer is present (Fernández et al. 1985; Lazo 1990; Mendez 1990; Barrios 1992; Gonzalez et al. 1993; Sponagel 1994). However, strains that yield high spore production, high pathogenicity and adequate shelf life remain a challenge for mass production and field application.

Production of *B. bassiana* spores can be achieved using different methodologies, which can be classified into low input and industrial technologies. However, most production of fungal spores worldwide is carried out using simple technologies that demand low inputs (Ferron 1978; Hussey and Tinsley 1981; Alves and Pereira 1989; Antía et al. 1992; Jones and Burges 1997). Most of the production of *B. bassiana* spores in Colombia for coffee berry borer biocontrol is done using a simple sterilization technique based on cooked rice placed inside bottles. The spores are mainly used for field spray applications (Posada 1993; Bustillo and Posada 1996). The spores are harvested by washing them out from the rice media with a 1 % oil-water suspension (Antía et al. 1992). This aqueous spores suspension must be used immediately after preparation to avoid spore germination. Additionally, spore longevity is short if they are kept in the bottles because high moisture content causes rapid loss of spore viability. Harvesting the spores from the bottles is time consuming and if the cultures produced in the bottles are going to be used to produce a spore powder, and drying the rice with spores is difficult (Posada 1993; Gonzalez et al. 1993).

Another methodology for spore production involves the use of fermenters and artificial media. The advantages of this technology are that spores are easily harvested and can be used to prepare formulations. In Colombia, some private companies have been trying to develop *B. bassiana* formulations as wettable powders and dispersible granules (Morales and Knauf 1994; Marin et al. 2000). The quality controls over those formulations are not very consistent, based on tests conducted by CENICAFÉ, Colombia's National Coffee Research Centre. Any *B. bassiana* product that is going to be used for the coffee growers to apply against *H. hampei* is continuously evaluated for quality, using CENICAFÉ approved methodology (Vélez et al. 1997; Marón et al. 2000).

Jenkins (1995) reviewed spore yields for 13 fungi produced on semi-solid media and found only one product that yielded concentrations per gram higher than 1 × 10^10^ spores. The product, based on *B. bassiana* that is sold by Laverlam International corporation www.laverlamintl.com (previously known as Mycotech) was reported to have 5.8 × 10^10^ spores per gram of substrate. This implies that the conversion of substrate to spores was high, and that at least some formulations already in the market should be able to increase yield while remaining economically viable to produce.

The development of mycoinsecticides to be sprayed with controlled droplet application technology must consider the production and formulation system. The spores produced for oil formulation must be lipophilic, this means that they should be produced using surface liquid media, on solid substrates or a combination of these methodologies that are called diphasic liquid—solid fermentation (Jenkins and Goettel 1997; Lomer et al. 1997). The spores that are produced on the media after it has dried can be harvested as a dry powder. Harvesting using fine sieve meshes prevents clumped spores passing through that can block nozzles and cause difficulties in obtaining an even distribution of the spores in the droplets. Also, harvesting with sieves allows the user to know the particle size, which is an important parameter in quality control.

The aim of this study was to produce *B. bassiana* spores using the diphasic liquid—solid fermentation technique developed for the LUBILOSA (Lutte Biologique contre les Locustes et Sauteriaux, www.lubilosa.org) project to produce *Metarhizium flavovinde* (Lomer et al. 1997). The production was carried out using two *B. bassiana* isolates that showed high virulence to the coffee berry borer. All the production steps were closely followed to record the variables related to time of harvest, substrate moisture content, method of harvest, particle size and quality control parameters such as: concentration, germination, pathogenicity, powder spores moisture content and purity. The spores harvested were used in subsequent formulation and field application experiments.

## Materials and Methods

### Isolates

Two isolates of *B. bassiana* were prepared: (1) *B. bassiana* 9002 was isolated from the coffee berry borer *H. hampei* (Coleoptera: Curculionidae) collected in Ancuya (Nariño) in a coffee plantation and (2) *B. bassiana* 9205 was isolated from the sugar cane borer, *Diatraea saccharalis* (Fabricius) (Lepidoptera: Pyralidae), collected in Palmira (Valle del Cauca, Colombia) in a sugar cane plantation. Both isolates were selected as the most virulent coffee berry borer strains in the CENICAFÉ *B. bassiana* collection.

### Production

Five trials were carried out, each one with a batch of 30 kg of *B. bassiana* cultured on rice using LUBILOSA's diphasic liquid—solid fermentation methodology (Jenkins, 1995) using plastic bags. Each bag contained 200 g of cooked rice inoculated with *B. bassiana* inoculum grown on liquid media. After the fungus culture had grown, spores were harvested as powder and then sieved through three sets sieves with mesh sizes of 18 (1 mm), 35 (500 m), and 60 (250 m). The spore powder, passing through all three sieves, was used for subsequent research such as spore storage in oil, spore oil formulation and feasibility of spraying using controlled droplet application technology.

### Experimental design

The experiment was set up as a completely randomized design with a factorial arrangement (2 × 4). The treatments were two isolates and four different times of harvest, 15, 25, 35 and 45 days after the inoculation and incubation. Each treatment combination was replicated five times and each replicate consisted of one bag of 200 g. The data were analyzed using one-way analysis of variance (ANOVA: SAS Institute 2003)

### Spore harvesting and drying

The spores were harvested at four different time intervals, 15, 25, 35 and 45 days after the inoculation, to evaluate if harvest time had any effect upon spore yield and to obtain an estimate of the optimal harvesting period. To harvest the spores as powder it was necessary to dry the fungus to reduce moisture content and allow the spores to separate from the rice substrata. The spores harvested following this procedure can be preserved for a long time without loss of germinative power or pathogenicity (Bateman 1995).

**Table 1.**
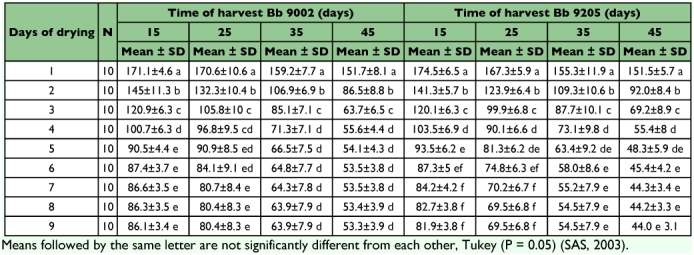
Weight (g) of the Beauveria bassiana culture and rice at different times of drying and before the spores were harvested.

Treatments were selected at random to be dried at each harvest time. To dry the cultures the plastic bags were opened in a room with a temperature of 15 ± 4°C and an average relative humidity of 55 ± 7 % and allowed to air dry. Harvesting was done both mechanically and manually for 20 minutes. The mechanical harvest involved the use of a shaker Ro-Tap Sieve Shaker (W.S. Tyler Inc., www.wstyler.com/) that uses horizontal circular motion and vertical taping motion to stratify and screen the particles. The manual harvest consisted of back and forth movements of the sieve. The samples were then put through 3 sieves as described above. The spore powder that was collected after sieving was weighed and kept in separate sterile vials for further assessments, such as moisture content and quality assessment (see below) and stored for later use inside a sealed silica gel vacuum desiccator and kept in a cool room under the conditions described above.

### Culture moisture content assessment

In order to monitor air drying of the rice cultures, each sample was weighed every day until the weight was stable. At the same time, from independent samples that were kept under the experimental conditions, sub samples were taken daily to assess the humidity content using oven drying where the sub samples were dried for 24 hours at 105°C (Rao et al. 2006). The aim of this procedure was to find out how many days it would take the fungus to be ready to harvest and also to determine the moisture content of the rice — fungus culture.

### Quality control of spores

The harvested spores were evaluated using a quality control methodology developed at CENICAFÉ which assessed spore concentration, germination, pathogenicity, purity, moisture content and particle size (Vélez et al. 1997).

**Figure 1.**
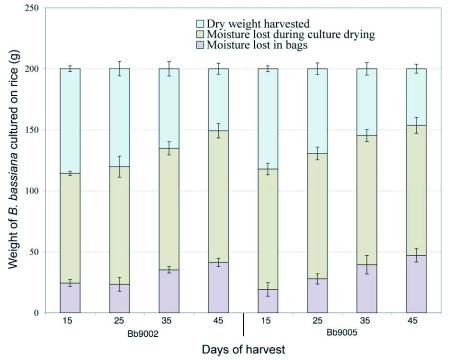
Weight loss by B. *bassiana* strains cultured on rice during oven drying following harvesting of spores. Bars represent confidence intervals for means (P = 0.05).

## Results

### Moisture content lost and dry weight of the B. bassiana culture on rice

Table 1 shows the results of moisture loss during air drying of rice substrate use to culture two *B. bassiana* isolates for spore production throughout four different harvest times and the final dry weight. Initially, the weight of the rice used as substrate plus *B. bassiana* liquid inoculum to start the fungus culture was 200 g wet weight per plastic bag and the weight was recorded just prior to first harvest. The daily moisture loss of the *B. bassiana* culture and rice shows a similar tendency for both isolates and was directly related to the harvest time. The moisture loss was greater the longer the time before spores were harvested. The moisture loss factor at each spore harvest was determined by doing a division between wet and dry cultures. The values of the factor obtained were 2.4, 2.4, 3.2 and 3.8 at 15, 25, 35 and 45 days respectively. These data estimate the amount of cooked rice and dry rice that need to be produced and harvested to obtain spore powder. These estimates were necessary so that appropriate quantities of spores could be produced for field experiments. At each harvest time the wet to dry factor was the same for 15 and 25 days and higher for 35 and 45 days. Analysis of variance of isolate, time of harvest and culture drying time were not statistically significant different for the interaction between factors (df = 24, F = 0.95, P = 0.5358). However, the statistical analysis (ANOVA) (Table 2) of independent factors showed significant differences. The analysis of variance of isolate and period of harvest per day that it took to dry the cultures were significantly different (df = 81, P = 0.0001). The significant differences were for isolates and time of harvest mainly between the first five days of drying cultures.

**Table 2.**
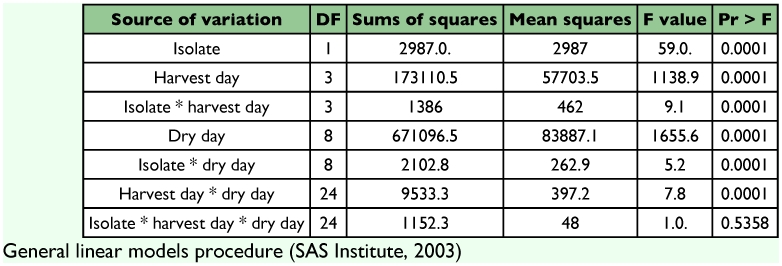
Analysis of variance for dry weight of *Beauveria bassiana* culture on rice to harvest the spores.

### Moisture content assessment of *B. bassiana* culture and rice before harvesting the spores

**Figure 2.**
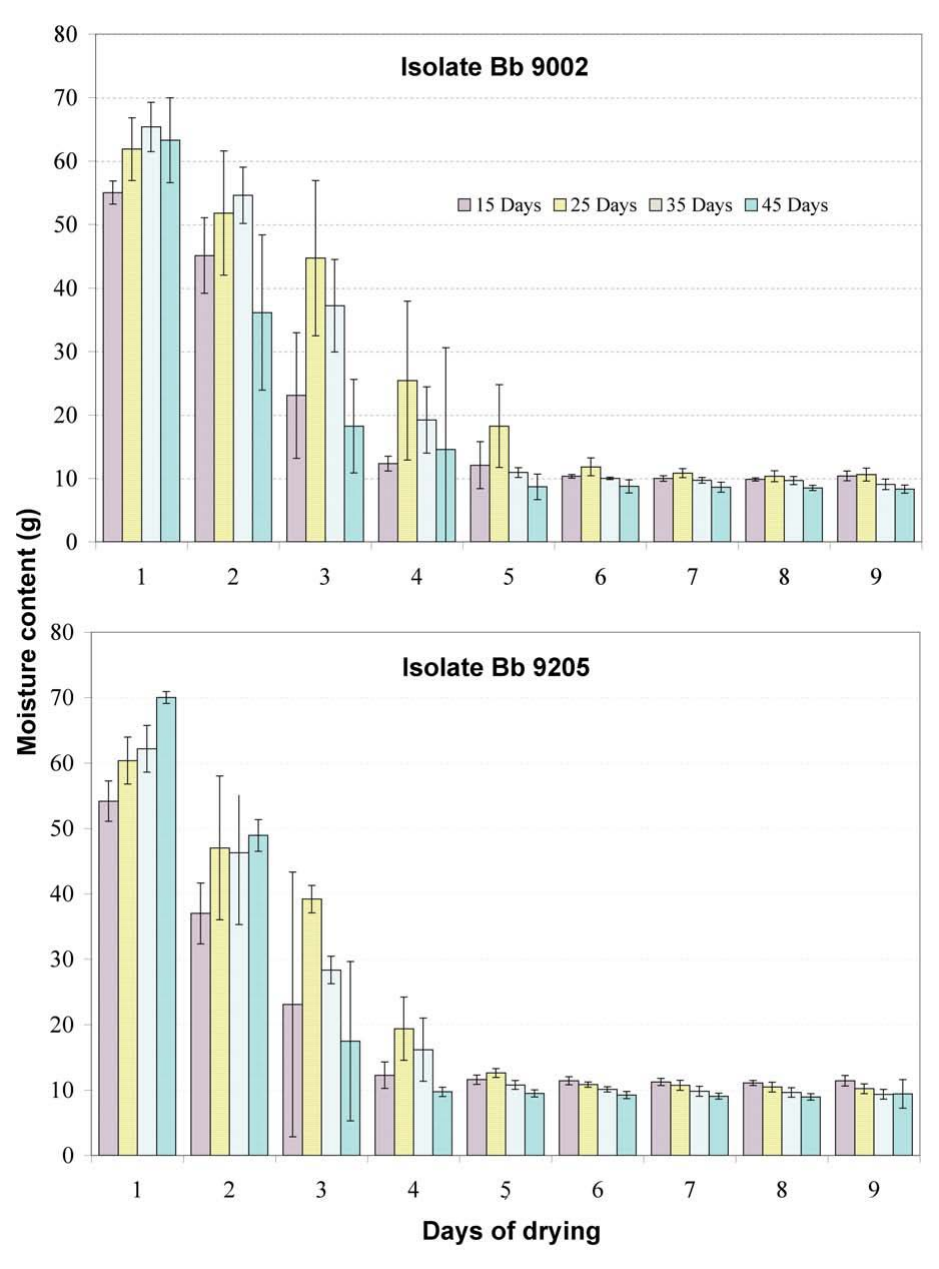
Loss of moisture content during air drying of two strains of *B. bassiana* cultured on rice at four different times previous to spore harvest. Bars represent confidence intervals for means (P = 0.05).

Figure 1 presents the moisture loss of *B. bassiana* culture and rice during air drying process before the spores were harvested. The *B. bassiana* culture and rice in to each treatment were dried for nine days at which point the moisture content was stable (Figure 2). The moisture content fell rapidly during the first four days and there was variation between days for different isolates and times of harvest (Table 1, Figure 2). After five days the moisture loss was relatively small and remained steady at around 10 % until the ninth day. This final moisture content was determined and monitored drying culture and rice subsamples daily in an oven in order to harvest the spores with the lowest moisture content.

### Quality control of harvested powder spores Spore powder weight

**Figure 3.**
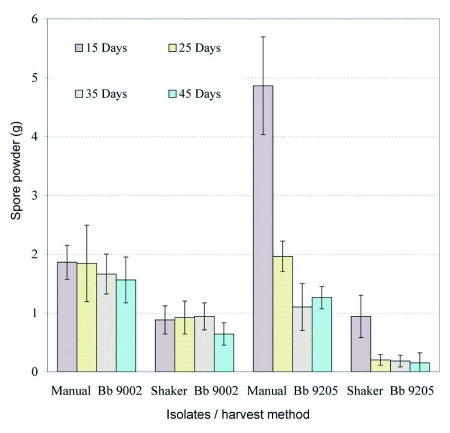
Weight of spore powder harvested from two strains of *B. bassiana* cultured on rice using two methods of harvest at four different times. Bars represent confidence intervals for means (P= 0.05).

Figure 3 shows the spore powder weight harvested from *B. bassiana* isolates using manual and mechanical methods at different times of harvest. The analysis of variance of isolate, method and time of harvest showed significant differences for factor interactions (df = 3, F = 24.7, P = 0.0001).

Spore powder production showed considerable variation. Production of both isolates was higher using the manual method compared with the mechanical method. The overall spore production by isolate shows that *B. bassiana* 9205 was slightly more productive than *B. bassiana* 9002 and the highest production of both isolates was obtained by manually harvesting the spores 15 days after inoculation.

### Spore powder production: concentration

**Figure 4.**
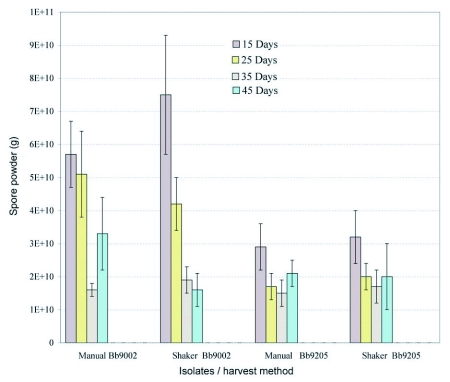
Spore production per gram of two isolates of *B. bassiana* cultured on rice harvested using two methods and four times after inoculation. Bars represent confidence intervals for means (P= 0.05).

Figure 4 shows the spore concentration produced by both isolates and harvesting methods estimated as grams of *B. bassiana* spore powder. The spore concentration was highly variable for harvest methods and for isolates. The spore concentration was higher for the mechanical method. This result was the opposite for the spore powder weight where a higher yield was obtained using the manual method.

Spore concentration was higher when harvested earlier compared with later times. It was observed that after harvesting the rice cultures *B. bassiana* spores remained on the rice. Following sieving these cultures were washed out with water plus 1% of emulsifiable oil to extract the spores for counting and to calculate the total concentration for each treatment.

Table 3 shows by isolate, time and method of harvest, the total production of spores estimated from the concentration of spore powder and spores remaining in the rice after harvest. The extraction of the remaining spores from the rice cultures showed that a high number of spores remain in the rice after sieving; the number of spores that remained on the rice was greater than the number of spores that were harvested as powder. The data show that *B. bassiana* 9205 is more productive than *B. bassiana* 9002. Production of *B. bassiana* 9205 was higher 15 days after inoculation and thereafter declined gradually to 45 days, which resembles a typical growth microbial curve. In contrast, *B. bassiana* 9002 did not show this pattern of spore production.

Although the total spore production of isolate *B. bassiana* 9205 was higher than *B. bassiana* 9002 (Figure 4, Table 3) the spore powder weight was higher for *B. bassiana* 9002, which was not desirable for formulation preparation because a high proportion of the powder was starch.

### Germination test

The germination test was conducted at 24 and 48 hours. The test was done for all harvest periods and isolates. Overall, the germination for all treatments was over 75% and germination at 24 hours was over 80%. Germination counts at 48 hours were difficult to perform because they were overgrown with mycelia and the risk of mistakes was higher. The germination tested at 24 hours for *B. bassiana* 9205 was higher after 25 days of harvest, while *B. bassiana* 9002 was higher after 35 and 45 days.

### Pathogenicity test

Table 4 shows the pathogenicity results of *B. bassiana* spores using the CENICAFÉ bioassay methodology (Vélez et al. 1997). The mortality of *H. hampei* was not significantly different between isolates and days of harvest (df = 3, F = 1.5, P = 0.2089) and was over 92 % for both isolates and for all different times of spores harvested.

The mean mortality time (MMT in Table 4) in days of *H. hampei* treated with the spores harvested in this experiment was not significantly different between isolates and days of harvest (df = 3, F = 0.97, P = 0.4077). Isolate *B. bassiana* 9002 at harvest times of 15, 25 and 45 days did not show a significant difference in mean mortality time, but between 15 days and the spores harvested 35 days after inoculation there was a significant difference (df = 156, F = 4.4, P = 0.0056). Isolate *B. bassiana* 9205 harvested at 15 and 25 days showed significant differences in mean mortality time compared with the spores harvested at 35 and 45 days. For both isolates, spores harvested at 15 days were more pathogenic to *H. hampei* than spores harvested after 35 days. The spores harvested at days 35 and 45 showed no significant differences between them (df= 156, F = 6.3, P = 0.0004).

### Purity

The presence of contaminant microorganisms was low, less than 2 % of colony-forming unit for both isolates and was present only in the spores harvested on day 25. Only the presence of contaminant fungi was detected. These were identified as *Penicillium* spp. and *Cladosporium* spp.

### Spore powder moisture content

The moisture content of *B. bassiana* spore powder harvested at different times showed a tendency to be lower for isolate 9002 and was higher for the spores harvest after 15 days. At this time 9205 had moisture content of 13 ± 3.1 % compared to 11.4 ± 0.5 % for isolate 9002. After harvest at 25 and 35 days the moisture content was similar for both isolates. At 45 days the moisture content dropped dramatically and was 6.3 ± 0.1 % for 9205 and 5.9 ± 0.9 % for 9002.

### Spore powder particle size

The spores harvested were taken from the bottom of a set of sieves of size 18 (1 mm), 35 (500 m) and 60 (250 m); therefore, the spore powder that was obtained had particle sizes of less than 250 m.

## Discussion

Moisture loss was a result of the metabolic activity of the fungus, transpiration and diffusion while the cultures were developing. Moisture was also lost when the cultures were dried for nine days in a cool room with 55 ± 7 % average relative humidity. The information obtained from drying suggests that the cultures can be dried in five days following the same process as in this study, which would save time and resources.

The lowest moisture content achieved from harvested spore powder was around 10% for the best harvest period (15 days). This moisture content, obtained under controlled conditions and after nine days of air drying was still too high to store the spores and preserve their viability. Ideally, a moisture content of 5% would have been preferable (Bateman 1995). One way to decrease the moisture content would be to keep the spore mix with a desiccant such as silica gel which would help to achieve the objective of spore storage with low moisture content (Moore and Caudwell 1997). The use of silica gel to maintain the spore moisture at a low level would not pose a technical problem even for larger scale production.

**Table 3.**
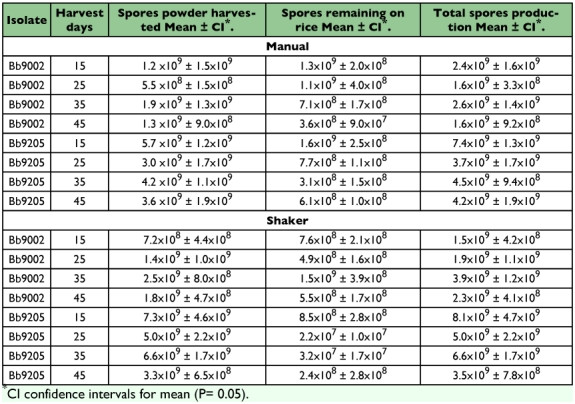
Concentration of spores of two strains of *B. bassiana* harvested as powder from rice cultures, spores remaining attached to dried rice after harvest and total estimate of spores production per gram.

Moisture content can influence the weight of the spores harvested. Although the spores with the highest weight also have high moisture content, this did not mean that they have high spore concentrations due to the presence of starch from the rice. Starch could block the nozzles of application equipment. These characteristics need to be evaluated if fungal production is to be scaled up to avoid application problems in the field situations. The powder harvested from *B. bassiana* cultures on rice is a mixture of starch and spores. The high weight of powder harvested from *B. bassiana* 9002, which had low spore concentration, means that there is a difference between *B. bassiana* isolates in the ratio of starch to fungal spores and this needs to be considered in mass production.

**Table 4.**
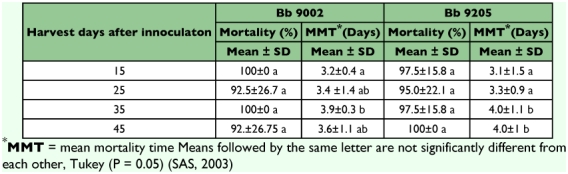
Pathogenicity of two strains of *Beauveria bassiana* against the coffee berry borer evaluated with spores harvested at four different times.

The harvest methods used showed differences in the amount of powder extracted from the cultures and the concentration of spores per gram of powder. The possible reason for this result was that the mechanical method used the same frequency during the time the cultures were being sieved, while in the manual method the rhythm of shaking changed more often because the operator became tired. Also manual shaking could be stronger than the mechanical method allowing more rice starch to pass through the sieve mesh thereby adding more weight to the spore powder.

It is not clear what happens with the spores, in terms of numbers, when the inoculation to harvest period is increased. It may be that they germinate and become mycelia, thereby, causing the loss of biomass. Alternatively cultures may begin to degrade because there are not enough resources to continue growing. To avoid a loss of spore production the harvest needs to be done early. Further experiments around 10–20 day intervals should be carried out to determine the optimum harvest period.

The results of this study show that if a *B. bassiana* spore production system is going to be based on rice, then a better method of harvesting the spores will be required to make the process more efficient because *B. bassiana* spores are strongly retained on the dry rice. Also, spore production may vary due to the different characteristics in strains, such as different patterns of spore production among various isolates.

The mechanical and manual harvesting methodologies used had low efficiency to extract the spores from the rice grain and this result combined with the low spore productivity (less than 1 × 10^10^ spores/g using the production methods evaluated in this study) require looking for better methods of *B. bassiana* mass production and spore harvesting if the fungus is going to be used as a mycoinsecticide (Ye et al. 2006; Bateman, 2007). Although spore production was improved over that obtained from culture in bottles (Posada 1993). Low yields below 1 × 10^10^ spores/g remain one of the major constraints in the production of reliable mycoinsecticidal products (Jenkins, 1995). If industrial production of entompathogenic fungal spores as a mycoinsecticide is planned it is necessary to adapt or change production methods to increase spore production and harvest with greater efficiency (Ye et al. 2006; Bateman, 2007). In the case of *B. bassiana* products based on rice, it will be necessary to improve the extraction method to remove the spores that are retained on the rice, which were twice the spores produced.

However, the production method for *B. bassiana* showed that high quality spores can be obtained according to the standard of purity established by CENICAFÉ (Vélez et al. 1997) and that these can be used as oil based formulation for control droplet application, even though this application technology requires very high single spore concentration (Bateman, 1995).

Additionally, the germination and pathogenicity of the spores, which are the most important quality control parameters, showed high values resulting in rapid mortality of coffee berry borer. The viability of the spores assessed for germination test at 24 hours was more accurate than when assayed after 48 hours. This implies that the assay of germination must be carried out within 24 hours as was also found by Bateman (1995). Proper evaluation of germination is an important part of determining the potential of spores for field use. Also the germination value obtained at this time is a parameter that needs to be considered independently of spore survival following their application.

The pathogenicity test showed mortality levels over 92.5% for both isolates and harvest times. The use of high quality standards for spores increases the likelihood of success when applied in the field. The mean mortality time was shorter for the spores harvested early than for the spores harvested later. A possible reason was that early harvested spores could have a higher proportion of viable spores and therefore they more promptly infected the beetles and caused more rapid mortality than spores harvested later. However, there is no evidence to support this hypothesis. The mean mortality for both isolates harvested times at 15 days were shorter and therefore both isolates caused high mortality in *H. hampei* compared to the other times of harvest.

The high spore quality, high spore germination, and high pathogenicity obtained using the methods used could be due to proper drying and harvesting of spores under controlled low temperature and relative humidity, which are factors recognized to cause a fast degradation of spore viability (Bateman, 1995).

The diphasic liquid—solid fermentation methodology is not suitable for *B. bassiana* powder spore production on an industrial scale because of its low efficiency and massive rice substrate requirement. This is illustrated in Table 5: to apply 5×1013 spores per hectare of 5000 cof- fee trees, 1×1010 spores per tree are needed. This would require 39.2 kg of dry cultures and 92.3 kg of cooked rice (wet weight). To spray 1000 ha would require 92,336 kg of cooked rice, which is clearly preposterous. Such a figure is also quite difficult to manage and scale up to industrial production, even in developing countries where labor is relatively inexpensive.

To carry out this production also requires laboratory facilities for cooking and sterilizing the rice, inoculum media, packing material and labor, a place to keep the culture while it develops, space and labor for spore harvesting, plus the hazardous risk to the workers exposed to airborne spores.

*B. bassiana* spore production needs to be highly efficient and productive to make a successful, inexpensive mycopesticide based solely on *B. bassiana* spores. Current diphasic liquid solid fermentation technologies are incapable of yielding mycoinsecticide concentrations higher than 1×10^10^ (Jenkins 1995). In addition to production constraints, entomopthogenic fungi have to compete against chemical insecticides and require several applications during the crop cycle (Yendol and Roberts 1970). They also need to be comparable in terms of efficacy, achieving mortality rates higher than 80 % under field conditions (Thomas et al. 1997).

The methodology evaluated in this study allows for the production of high quality *B. bassiana* spores, but the quantities produced are only suitable for small-scale laboratory and field trials. All the constraints analyzed above do not imply that the use of *B. bassiana* against the coffee berry borer should be stopped. In addition to this fungus being the most important natural enemy of the coffee berry borer (Ruíz 1996), field studies using *B. bassiana* and have shown that it is a promising strategy and a key component of the integrated pest management of the coffee berry borer even though they are in their initial stages (Posada 1998; De la Rosa et al. 2000; Haraprasad et al. 2001; Posada et al. 2004). The use of entomopathogenic fungi is a technology that is still being developed and improvements in production, formulation and field application are needed. There are several alternatives for using the fungus against the coffee berry borer, such as “inoculum-introduction” or “augmentative -inoculative strategies” (Hajek 1993) that can contribute to an epizootic condition and create a permanent mortality factor in the field.

**Table 5.**
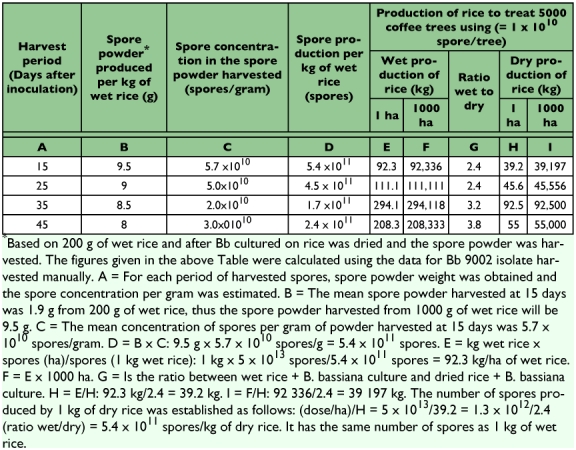
Relationship between time of harvest, spores produced by *Beauveria bassiana* 9002 cultured on wet rice, spore concentration and the production of wet and dry rice necessary to treat a number of coffee trees and hectares.
